# Considerations, Caveats, and Suggestions for the Use of Polygenic Scores for Social and Behavioral Traits

**DOI:** 10.1007/s10519-023-10162-x

**Published:** 2023-10-06

**Authors:** Amy L. Non, Jessica P. Cerdeña

**Affiliations:** 1 Department of Anthropology, University of California San Diego, La Jolla, CA USA; 2 Institute for Collaboration on Health, Intervention, and Policy (InCHIP), University of Connecticut, Storrs, CT USA; 3 Department of Anthropology, University of Connecticut, Storrs, CT USA; 4 Department of Family Medicine, Middlesex Health, Middletown, CT USA

**Keywords:** Polygenic risk scores, Risk scores, PRS, Behavioral genetics, Genome-wide association

## Abstract

Polygenic scores (PGS) are increasingly being used for prediction of social and behavioral traits, but suffer from many methodological, theoretical, and ethical concerns that profoundly limit their value. Primarily, these scores are derived from statistical correlations, carrying no inherent biological meaning, and thus may capture indirect effects. Further, the performance of these scores depends upon the diversity of the reference populations and the genomic panels from which they were derived, which consistently underrepresent minoritized populations, leading to poor fit when applied to diverse groups. There is also inherent danger of eugenic applications for the information gained from these scores, and general risk of misunderstandings that could lead to stigmatization for underrepresented groups. We urge extreme caution in use of PGS particularly for social/behavioral outcomes fraught for misinterpretation, with potential harm for the minoritized groups least likely to benefit from their use.

## Introduction

Developing polygenic scores (PGS) is a rapidly growing approach in biomedical and behavioral sciences. PGS (aka polygenic risk scores, PRS, or risk scores), compile information across hundreds to thousands of genetic variants into a single score to estimate an individual’s genetic risk for a complex trait. These are typically calculated as a sum of all genetic risk alleles that associate with a particular trait in a reference population, weighted by the effect size estimate (Choi et al. 2020). Because costs of sequencing have fallen so dramatically in recent decades, scientists can now generate these scores based on data from millions of people who participate in genome-wide association studies (GWAS), including the multitiudes who send their DNA samples to large direct-to-consumer genomics companies such as 23andMe, and large medical studies. Such large datasets allow for the precise detection of very small genetic effects. However, they also suffer from several limitations in their design and interpretation that reduce their value, particularly in the realm of behavioral genetics.

In this essay, we call attention to multiple concerns with the increasing use of PGS in behavioral genetics. We argue that since these scores are derived from statistical correlations, they carry no directly causal genetic information and require no understanding of the biological role of underlying genes in contributing to any trait or disease. Thus, although the scores may have statistical significance, they may entirely lack biological meaning and their therapeutic or research potential may be empiric rather than targeted. Moreover, these scores depend on the diversity of the reference populations and the genomic panels from which they were derived, which often do not fit well with the populations to which they are applied. We further highlight social and ethical limitations that should be considered when using PGS, particularly for social and behavioral traits.

### PGS in Behavioral Genetics

**Fig. 1:**
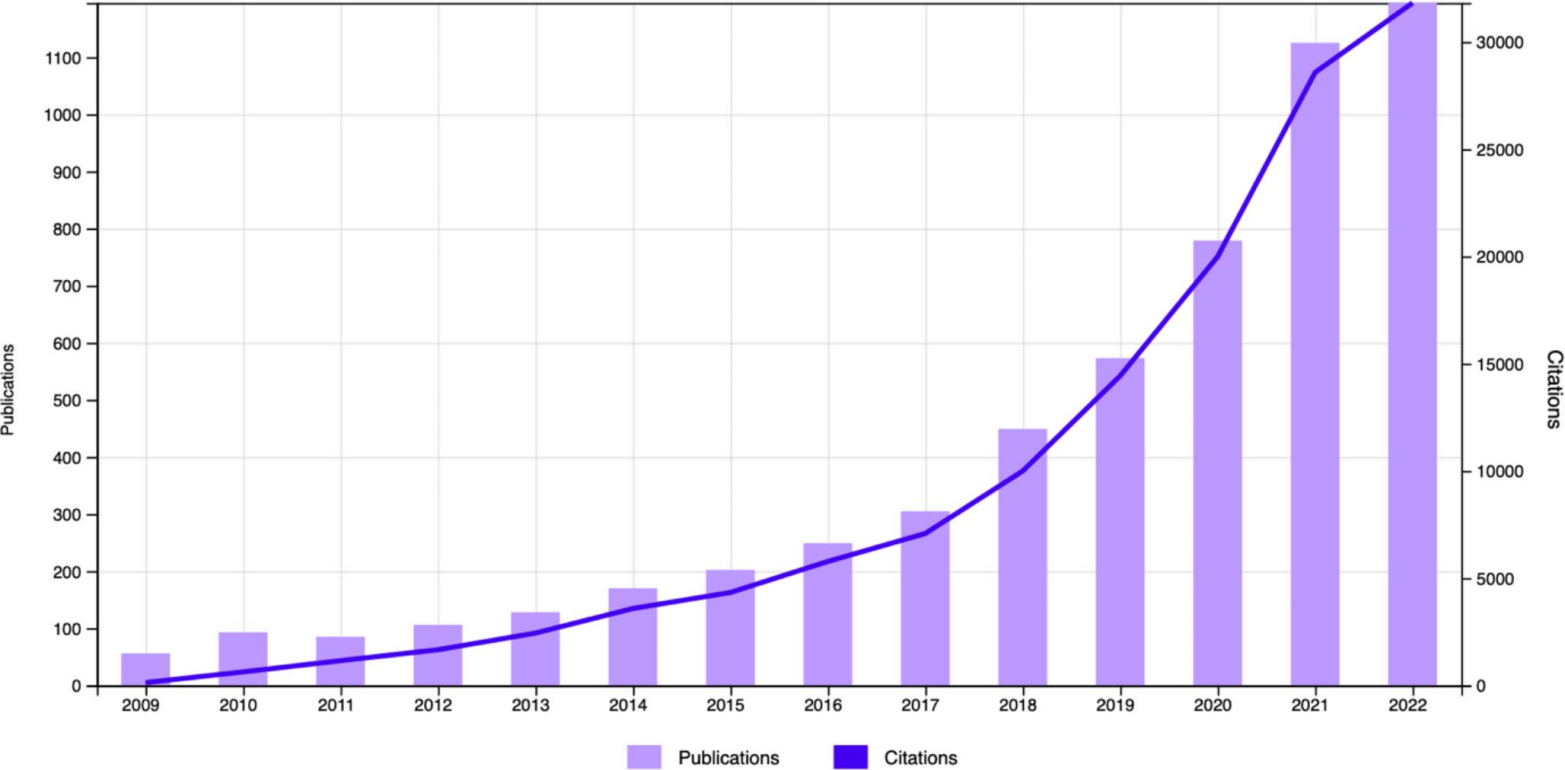
Publications (purple bars) and citations (blue line) demonstrating increasing references to polygenic scores in abstracts of articles in Web of Science between 2009 and 2022

Since its first development in 2009, use of PGS has become increasingly widespread to estimate risk of complex diseases, and has especially exploded in the realm of predicting social and behavioral traits. In a literature search of Web of Science conducted by Plomin and von Strumm 2021, they identified over 1000 publications using the terms ‘polygenic score’ OR ‘polygenic risk score’ OR ‘polygenic risk,’ by early 2021. In an update to this search using the same terms identified in the abstracts but extending just to the end of 2022, we identified over 5,516 publications since 2009, with over 78,993 citing articles (Fig. 1). The bulk of these papers were classified in Web of Science Categories for Psychiatry/Psychology/Behavioral traits (32.1%) followed by Genetics-Heredity (18.4%), with the remaining under various medical subfields, highlighting the intense focus on PGS for social, behavioral, and psychological traits.

Social scientists as diverse as economists, sociologists, and behavioral psychologists alongside geneticists have been developing PGS for traits as wide-ranging as loneliness (Day et al. 2018), smoking behaviors (Belsky et al. 2013; Chen et al. 2018), subjective well-being (Patel et al. 2021), parental caregiving (Wertz et al. 2019), cognitive measures of verbal and spatial reasoning (Liu et al. 2020), and most commonly in recent years, educational attainment (EA) (Rietveld et al. 2013; Lee et al. 2018; Okbay et al. 2022a). Although it may seem counter-intuitive to imagine how a genetic score can predict variables so obviously social as “loneliness,” a person’s genome indeed influences many relevant factors that contribute to these social traits. These include subtle personality characteristics (e.g., diligence, patience), health factors (e.g., chronic illnesses), and behaviors (e.g., sleeping patterns, addictive drug use). Thus, PGS capture many of these indirect traits, which associate with dozens of different outcomes, rather than strictly the one under study. In fact, the genetic predictors of educational attainment, one of the most well-studied behavioral traits, has shown to correlate with many other outcomes including height, parenting, antisocial behavior (i.e., criminality), and healthy aging, to name a few (Kong et al. 2018; Wertz et al. 2018, 2019; Wu et al. 2020; Schork et al. 2022). Moreover, the majority of PGS only estimate very small contributions of the total variance in these traits (e.g., 11-13% of educational outcomes (Okbay et al. 2022b), leaving the vast majority (87–89%) unexplained. Although these levels of genetic prediction may be most useful at the extremes of the PGS distribution (e.g., 75% of those in the highest decile of polygenic risk score for education go to college (Plomin and von Stumm 2022), the majority of people fall in between, where the predictive ability is less powerful.

### Methodological Considerations

Many methodological considerations can bias PGS estimates, particularly when examining traits in diverse populations. The first is that the predictive power of a particular PGS in a specific population depends on the appropriateness of the original single nucleotide polymorphism (SNP) panel used to develop the score. Importantly, ascertainment bias in the SNP arrays used to generate the GWAS data from which these scores were originally derived leads to the underrepresentation of rare alleles, especially from the most diverse populations of Africa. Thus, it is important to move beyond commercial SNP arrays to much more expensive whole genome sequencing or improve the design of arrays to include many more diverse variants (De La Vega and Bustamante 2018). Additionally, the lack of diversity among most participants of GWAS – over 80% are of European descent (Sirugo et al. 2019) – means that the training populations used to identify relevant alleles to build these scores do not reflect the genetic variability of the global human population. As a result, the predictive accuracy of the scores outside European groups is much lower. This is in part because allele frequencies differ across populations, such that more frequent alleles in the training set may be entirely lacking from the target population, and vice versa. Additionally, patterns of linkage disequilibrium arising from different demographic histories vary across ancestry groups, which can alter the estimated effect size of alleles in different populations. Finally, the effect size of each allele may differ across populations if its effect on a phenotype depends on interaction with variation in the surrounding genome (Mostafavi et al. 2020), or in interaction with different environments. Accordingly, the scores systematically perform best with target populations of European ancestry, and concerningly misestimate outcomes in unpredictable ways in other groups (Martin et al. 2017, 2019a; Kim et al. 2018). For example, the educational attainment PGS accounts for only 0.2–2.3% of the variance of education in those of African ancestry in the US or UK, relative to 13% for those of European ancestry (Duncan et al. 2019). Similarly in a PGS study of schizophrenia, the risk scores were decreased in Africans relative to all other populations, despite the fact that similar disease prevalence has been observed across populations (Martin et al. 2017). This bias is exacerbated by the use of direct-to-consumer genetic databases and opt-in biobanks as giant discovery datasets, as their members are not representative of the nation, being generally more highly educated, wealthier, and healthier than the population average (Fry et al. 2017; Uffelman et al. 2021).

This problem can be generalized beyond ancestry effects, as any factors that differ between the training and target samples, such as age, location, socioeconomic status, or cohort effects (e.g., birth year) can reduce the accuracy of the PGS (Choi et al. 2020). For example, in one study testing the replicability of PGS on cardiometabolic traits, the effect of birth year was substantial, supporting the role of changing environmental and demographic factors over time (Loika et al. 2020). In another study, using over 300,000 White British individuals in the UK Biobank, researchers demonstrated that *even within the same ancestry group*, the prediction accuracy of the PGS (measured by incremental R^2^) for diastolic blood pressure depended on the sex ratio of the training dataset; for BMI, it depended on the age range of the training set; and for years of education, it depended on the socioeconomic status (SES) of the training set (specifically 2-fold higher accuracy in the lowest SES quartile compared to highest, when GWAS was performed in those of lowest SES) (Mostafavi et al. 2020). This study concluded that the environmental variance around these traits was not even the biggest problem for prediction accuracy, but rather more problems stemmed from the difference in magnitude of genetic effects among groups, indirect effects, and assortative mating. An important implication is that even after controlling for ancestry, PGS will not perform well across groups that differ largely in factors such as SES or other unknown confounders.

Failure of PGS to transfer well across populations also stems from the general failure of the underlying GWAS findings to replicate (Ioannidis 2007). While GWAS replicability has improved in recent years as study sample sizes have dramatically increased (Marigorta et al. 2018), they still typically underperform in sensitivity (ability to detect true positive results) for complex diseases and traits; e.g. the predictive power area under the ROC curve is < 0.7 for most studied traits (So and Sham 2017). Sample sizes in the millions can improve the predictive power of PGS, but larger samples also introduce more heterogeneity in phenotypes and genotypes adding more challenges. Even if all true effects of causal variants could be identified, the degree of variance one can explain with a PGS will always be limited by the true heritability of the trait under study (Marigorta et al. 2018), which is likely smaller for complex social or behavioral traits than most diseases, as they have large environmental components. Another factor complicating replication is sparse genotyping approaches, such as array-based designs, which are the most cost-effective but require genetic imputation to infer the full set of genotypes in a PGS estimate. One study found that imputation introduces minor changes in PGS, but in some rare instances can result in a dramatic change to the score which can be very problematic at the individual level if PGS are used in healthcare settings (Chen et al. 2020). While PGS is not currently being used in clinical consultations, they have the potential to be used for clinical risk assessments, which is prone to all the problems of replicability and transferability previously noted. If the current scores were used in clinical application, they may perform well when stratifying by disease risk, but they would in fact underdiagnose the most vulnerable individuals coming from the populations least represented in the genetic studies.

Another major challenge in developing PGS—particularly for complex social/behavioral traits—is the difficulty in operationalizing these very complex multifactorial traits into a straightforward quantitative measure. Educational attainment has been an attractive outcome to model given how simple it is to sum years of education; however, this crude measure fails to address variation in the quality of study, degrees obtained, social status achieved, or diminishing returns of education gains for certain racial groups (Assari 2017). Likewise, loneliness can be influenced not just by a person’s frequency of contact with social connections, but their perception and personality which influence how they report this measure. Similarly, psychiatric disorders such as depression and anxiety tend to present as more of a spectrum of phenotypes with enormous variation than an easily defined disease, often with overlapping traits across disorders, and overlapping symptoms even among cases and controls (Geschwind and Flint 2015). As such, the typical shortcut summary measures of very complex behavioral traits compound the imprecision inherent in PGS estimates.

### Theoretical Considerations

PGS also demonstrate considerable theoretical shortcomings. First, because the scientific community is still learning the basic functions of the human genome, no driving hypotheses guide the models that test associations between SNPs and outcomes of interest. A telomere-to-telomere genomic sequence, including repeat DNA sequences, was only completed in 2022 and functional genomics studies have not kept pace with sequencing efforts (Nurk et al. 2022). Due to linkage disequilibrium, correlations identified may or may not be ‘true’ as neighboring genetic variants tend to be inherited together through co-segregation during meiotic recombination. Phenotypes of interest may involve particular cell types, which differentiate through epigenetic processes that are not assessed in GWAS. Over 90% of genetic variants involve non-coding, regulatory elements, which may obfuscate their role in PGS (Cano-Gamez and Trynka 2020). Even though it is widely accepted that phenotypes frequently result from multiple loci that may themselves contain several genes (i.e., gene-gene interactions, or epistasis), PGS are not built from explanatory models to account for these relationships.

Second, purported genetic associations may be spurious, particularly for complex social and behavioral phenotypes. Most complex traits do not exhibit genetically deterministic patterns and instead vary considerably depending on environmental contexts, which are often not adequately measured or controlled in GWAS studies on which PGS are based (Martin et al. 2019b). This is particularly problematic when considering the lack of underlying socio-ecological diversity in most GWAS studies. For instance, in the latest GWAS of EA the effect of the PGS vanished after adjusting for the PGSs of relatives (Okbay et al. 2022a; Schork et al. 2022). This suggests that the PGS is acting primarily through indirect pathways, either as a result of assortative mating (parents of high education seek each other), parental behaviors (also influenced by the genome), population stratification, or health status—which together likely explain much of the heritability of EA.

Third, the conceptualization and operationalization of traits deployed in PGS often lack consistency. For instance, multiple studies assess “resilience” to complex social experiences including trauma, “victimization,” and bullying. However, definitions of “resilience” may be absent (Armitage et al. 2022), elaborate (Hess et al. 2021), or refer to entirely different concepts ranging from the absence of pathology to an adolescent trait (Docherty et al. 2018). Clinical terms like “susceptibility” may be used solely based on genetic estimates, promoting misunderstanding. Further, measures of the same environmental exposures vary widely. A systematic review including 17 studies of PGS for schizophrenia in people with experiences of childhood adversity analyzed 18 different measures of childhood adversity, only a few of which included validated instruments in the original studies (Woolway et al. 2022).

It seems reasonable to ask, does it matter if the alleles in the scores are directly causally relevant if the scores are generally predictive of the trait? We argue that much of the purported value of PGS, particularly for social traits such as EA, is invalidated if the alleles are not causally related. For example, if PGS for EA were used as a tool to generate specific hypotheses such as a list of cognitive-related genes or pathways to examine in functional follow-up studies (as suggested by Lee et al. 2018), they would be entirely inappropriate if the alleles are in fact related to parenting behavior or health rather than cognitive ability. Another potential use suggested by EA researchers (Lee et al. 2018; Okbay et al. 2022a) and others (Harden 2021) is to use the scores to target interventions, such as to enhance educational opportunities for those most likely to benefit, or alternatively for those most in need. However, when taking into account environmental context such as childhood SES, Belsky et al. (2018) discovered that the PGS for EA was much more predictive of social mobility (higher income in adulthood) for those with low relative to higher childhood SES. These findings suggest that PGS on their own have limited predictive power for social outcomes and may only be informative after taking relevant environmental factors into account. Interventions based on these scores could unfairly limit opportunities to those with the most supportive child environments, falsely assuming these children to have genomes most likely to benefit from attaining high educational achievement. Inversely, if interventions only target those most at risk based on the PGS score, they may fail if they do not account for the effects of an adverse child environment. In the end, child environments are likely more important than the genetic score in determining educational success, and do not require an expensive GWAS to measure.

### Social and Ethical Considerations

Perhaps most concerningly, PGS perpetrate ethical and social harms. Interpretations of PGS are often overhyped or misunderstood, promoting eugenicist beliefs. For instance, a 2022 study that tested associations between 33 PGS and fertility among 409,629 British participants of European descent across two generations found that scores predicting higher earnings, education, and health also predicted lower fertility (Hugh-Jones and Abdellaoui 2022), which very closely matched the language of late 19th-century British eugenicists. The authors applied a natural selection argument to these findings without presenting empirical evidence that the many genes involved in these scores exhibited selective pressures. These findings translated into a headline in *The Telegraph* suggesting, “Britons are evolving to be poorer and less well-educated,” demonstrating how reverse application of evolutionary theory to findings and oversimplified results can advance misunderstanding among lay audiences (Knapton 2022). PGS can also harden notions of racial essentialism, or the false belief in inherent (i.e., genetic) differences between people socially and politically organized into different racial groups. White supremacists have deployed PGS to make claims about the genetic superiority of White Europeans with respect to intelligence, brain volume, and educational potential on online forums (Stormfront.org 2023). Sociologist of science Aaron Panofsky identified especially problematic dialogue around PGS in the Pseudoscience journal group *OpenPsych*, which features contributing authors without professional credentials with eugenicist bents, including claims that PGS among Jewish people conferred advantages in cognitive ability and educational achievement (Panofsky et al. 2021). Beyond the potential misuse of PGS data for eugenicist rhetoric, PGS are currently being used in embryo selection, according to recent reports (Turley et al. 2021). This is an exceedingly dangerous trend, given the inherent problems with racial/environmental bias in PGS training sets discussed above, in addition to the risk that PGS may be capturing unmeasured confounders, rather than the outcomes of interest. We urge regulation and oversight to prevent PGS from being used in this harmful eugenics context.

We acknowledge that some PGS may be valuable for informing clinical interventions for the cumulative impact of variants that together confer large deleterious effects in health, as with certain determinants of cardiovascular disease (Aragam and Natarajan 2020; Klarin and Natarajan 2022). However, to date, most PGS have limited clinical utility. The ability of genomic scores to model disease risk is minimal, and requires consideration of other biomarker assessments and individual and environmental factors (Moorthie et al. 2022). Further, because many clinicians are not appropriately trained in high-throughput genomic analyses, healthcare providers are not equipped to interpret PGS in routine evaluations. If PGS are ultimately used in clinical settings, they have the potential to exacerbate health disparities given how they consistently perform vastly better in European populations relative to all other groups, unlike most other clinical biomarkers or diagnostic tools (Martin et al. 2019a), and are more accessible to wealthier populations.

### Suggestions for Future Research

Given the high enthusiasm and likely continued use of PGS for behavioral genetic traits, we provide a few suggestions for researchers to avoid these common pitfalls. First and foremost, we encourage thoughtful, theory-driven hypothesis testing and advise researchers to exercise extreme caution when determining PGS for traits that have predominantly environmental drivers and thereby high risk for misinterpretation and unintended consequences. For instance, though data availability may make it possible to determine PGS for outcomes such as unemployment and receiving supplemental nutritional or economic assistance, these are prone to ableist and racist readings and we believe the significant risks of calculating PGS in these cases outweighs any potential benefit. Second, as we have explained elsewhere (Cerdeña et al. 2022a, b), we encourage increased attention to and measurement of structural environmental factors that impact the development of social and behavioral traits. Third, when PGS are used, we suggest researchers ensure they communicate *within the text of their manuscripts* the caveats and limitations of their PGS score (not just in supplemental documents) to avoid misinterpretations. This includes specifying clearly to which populations these scores should and should not be applied, particularly if the samples used to derive them are not globally representative. Researchers should also ensure they clearly communicate the lack of evidence for causal relationships with genetic markers identified to ensure the scores are not prematurely used clinically or ever used for selecting embryos, or other eugenic uses. Finally, we recommend that PGS be validated in independent datasets, tested against validated biomarkers, and shown to be predictive of disease or disease progression before they are used clinically.

We believe PGS can be used responsibly for certain purposes. Assuming improvements are made in diversity of populations used to develop the scores, and that accurate scores can be developed with direct causal relevance to disease outcomes, we see potential value in PGS for revealing genetic variants and pathways of interest for diseases that can be validated in functional studies. For example, the American Heart Association has offered provisional guidance on the use of PGS in clinical practice, suggesting incremental predictive capability only for atrial fibrillation in individuals requiring close surveillance (O’Sullivan et al. 2022). PGS may also be valuable for identifying relevant variants to test in studies of gene-by-environment interactions. Clinically, they could have value in the behavioral realm in early screening for psychiatric diseases risks such as schizophrenia, for which PGS have one of the highest predictive powers relative to a range of diverse diseases (So and Sham 2017), in concert with repeated symptomatic screening and comprehensive family history data.

## Conclusion

In the realm of behavioral and social traits that are surely determined primarily by social forces, like educational attainment, PGS are of uncertain utility with serious concern about whether they could ever be ethically applied. How it would benefit anyone to know their educational attainment PGS is unclear, and contrary to some claims (e.g. Harden 2021), in our view, such scores are more likely to be used to create institutions that harden social stratifications than to soften them. For example, if we imagine separate schools for children with different PGS scores, it is more likely that more resources will flow to the schools with high educational attainment PGS than to the schools of those with low PGS scores. If certain adolescents are considered at high genetic risk for “externalizing behaviors”—including people of “African ancestry”—how soon will it be before these children are cast apart from peers and even more harmfully stereotyped? Given all of these inherent methodological, theoretical, and ethical concerns, we urge extreme caution in use of polygenic scores, particularly for social/behavioral outcomes fraught for misinterpretation and at risk of stigmatizing effects. In sum, if these scores are simply predictive but not necessarily etiologically relevant, often fail to replicate, and can be less predictive than simply asking a patient to report lifestyle or behavioral factors, we question if the value gained by these genetic scores is greater than the potential cost, both financially and in risk of misuse and misinterpretation.
